# Surgery

**Published:** 1859-01

**Authors:** 


### Bi-Monthly Periscope.

### SURGERY.

1. ON THE THEORY OF THE PRODUCTION OF HERNIA. By *Professor Roser.*—The views here advanced have already been published by Professor Roser, seventeen years since; but as they have not excited the attention he believes they deserve, he reproduces them with the advantage of being able to add, that all subsequent investigation during so long a period of an active career, has only confirmed his conviction of their truth.

Debating with a celebrated professor of surgery, he declared that a sudden production of a hernia was impossible, according to the laws of mechanics. The professor maintained the possibility, inasmuch as he had examined persons who had shown no signs of hernia, and yet, after violent exertion, its presence had become manifest. This, Roser regards not as observation, but as a *post-hoc* conclusion. It is well-known how difficult (or when small, impossible) it is to detect an empty hernial sac. We only recognize it when the intestine has entered it, and we can feel the impulse on coughing, etc.; but when such entrance is prevented by various circumstances, the most we can say is, not that no hernial sac, but that no hernia, is present. Why is it not possible, as Scarpa and Cloquet have shown, to produce a hernial sac on the dead body by the use of violent compressing power, aided by apertures made in the tendinous walls of the abdomen? First, because the peritoneum is not sufficiently elastic to bear the necessary extension; and, secondly, because compression of the soft and fluid contents of the abdomen acts by hydrostatic law; and although the peritoneum may be stretched, it is not thrust out as it is found to be in hernia, and as it may be to some extent by the finger locally applied.

Professor Roser advances these two propositions, that femoral hernia arises from the dragging out (*Herauszerrung*) of the peritoneum, and that external inguinal hernia, or more properly its sac, is almost always congenital. First, with respect to *femoral hernia*, the dragging out of the peritoneum is brought about by nodules of fat, which, appertaining to the sub-serous tissue, are firmly attached to the peritoneum. These nodules slide between the fibres of the *septum crurale*, thrust them asunder, and lead to their disappearance. The anterior part of the nodule passes out under the plica, covered only by *fascia superficialis*, and increases in size. Its movements are favored by its pyriform shape, and by the motions of the body, and the peritoneum following it, a sac is gradually formed. In all the instances of commencing hernia the author has had the opportunity of examining, he has found such fatty nodule at the apex of the sac. We cannot expect to find this in old or large herniæ, as it would disappear under pressure. Professor Linhart of Würzburg, the only anatomist who has of late years investigated the subject of hernia, has confirmed these views, stating that he believes that traction exerted on the peritoneum exerts far more influence in the formation of hernia than the pressure exerted by the contents of the abdomen.

*External Inguinal Hernia.*—The valvular character and oblique course of the inguinal canal, should have formed a sufficient reason for rejecting the ordinary theory of the formation of this hernia. All subsequent investigation has convinced Professor Roser that his statement made long ago, that this hernia is almost always congenital, is correct. He has found that the *hernia vaginalis funiculi*—*i.e.* an open state of the upper part of the vaginal process—occurs much oftener than is supposed. It has been found in almost all the children the subjects of inguinal hernia, whom he has examined; and he has frequently met in adults hernial sacs so long and narrow, that they could only be regarded as incompletely developed vaginal processes of the peritoneum, into which intestine had not passed. He has also found the anatomical signs, detailed in his former treatise, exhibiting the congenital nature of the affection. Other coexisting anomalies of the peritoneal formation are often met with. Besides the descent of the testis, there is a descent of the cœcum and sigmoid flexure, and disturbances of these often occur at the same time. As the author has found in almost all the outer inguinal herniæ that he has examined, such grounds for considering them congenital, he has come to the conclusion that the bulk of cases regarded as accidental do not merit the appellation, inasmuch as the sac has been in existence prior to birth. He refers in confirmation of his views to Camper's statement, that of sixty-three full-timed children in whom the testis had descended, the vaginal canal was obliterated only in seven, it being open on both sides in thirty-four, on the right side alone in fourteen, and on the left side in eight. So, likewise, Professor Engle, whose investigations are now publishing in the *Wien Wochenschrift*, states that in children at birth, or during the first fourteen days afterwards, the vaginal canal is found oftener obliterated, or at least considerably shorter, on the left than on the right side—a fact agreeing with the preponderence of hernia on the right side. He found the canal entirely closed at birth in ten per cent. After fourteen days no trace of it could be found on the left side in thirty per cent., while it remained open on both sides at the end of fourteen days in sixty per cent. In the adult the presence or the remains of the vaginal canal was observed in thirty-one per cent. of the bodies examined, on both sides in 37·5 of these, and on the right side alone in 62·5.

*Outer Inguinal Hernia in the Female.*—The author long since proposed the question to Professor Meyer of Zurich, whether women were not liable to a similar descent of the peritoneum as men; and that writer, in a paper in Müller's *Archiv*, has shown that in the female as well as the male fœtus, a projection of the peritoneum into the inguinal canal does take place. Its metamorphosis, however, occurs much earlier, as soon as the fifth month; and it is also less considerable, and therefore less liable to disturbance than in the male sex.

*Internal Inguinal Hernia.*—This, Professor Roser observes, may, in some cases, have a fatty origin, like femoral hernia; but he believes that other cases arise from a local protrusion of the peritoneum, in aged and relaxed subjects, in whom a partial atrophy of the *fascia transversalis* has taken place. This is a very different thing, however, to the sudden production of hernia usually admitted. He believes with respect to internal inguinal hernia, that the fact that it occurs almost exclusively in aged men is not generally known. It takes place indeed much oftener than most authors admit; and may be almost said to be as frequent in old men as femoral hernia is in old women. The much greater narrowness of the ring is the reason it does not occur often in old women.

*Umbilical Hernia.*—This is the only hernia which is produced according to the old theory, viz., by a *vis a tergo*, and even here the author suspects that in some cases it may arise from a congenital protrusion of the peritoneum, remaining from the fœtal state. As this hernia differs in the mechanism of its formation from other kinds of hernia, so does it in the remarkable fact of its being generally spontaneously curable, as may be seen from the small number of cases met with in the adult, compared with the large number occurring in infancy. In aged and fat individuals, accidental hernia of this form is, however, frequent.

Originally commencing his investigation with a purely scientific object, Professor Roser has since found that it has an application to legal medicine, the question not unfrequently arising, whether a hernia has been produced in consequence of acts of violence in scuffles, assaults, &c., and the culprit risking to be dealt with too harshly in consequence of the off-hand way in which the affirmative is pronounced.—*Arch. für Physiol. Heilk.*, 1858, pp. 60–70. (*British and Foreign Medico-Chirurgical Review*, Oct. 1858.)

2. SYPHILIZATION; ITS MODE OF PERFORMANCE AND ITS RESULTS. By *William Bœck, Professor of Medicine in the University of Christiania, etc.*—M. Auzias Turenne, as early as the year 1844, made various attempts to transfer syphilis from man to the lower animals; at length he succeeded, and by repeating his experiments in the same animals, he observed that they at length became inured to the syphilitic virus. He called the condition so obtained "syphilization;" and on the 18th November, 1850, sent a memoir detailing his observations to the Academie des Sciences. M. Sperino of Turin, immediately made use of the discovery of Auzias Turenne, for the purpose of curing syphilis by continued inoculations. A great many successful experiments, commenced in the month of January, 1851, convinced him of the possibility of its efficacy for the purpose in question;* and, on the 23d of May, 1851, he laid his first communication upon the subject of syphilization before the Medico-Chirurgical Academy of Turin. In the same year, I was traveling through the northern part of Italy; I did not, however, visit Turin; but in Milan I heard a great deal about the successful experiments of M. Sperino.

As long experience had taught me the uncertainty of the treatment hitherto directed against syphilis, I believed it not only allowable, but even a duty, to give a trial to a new remedy, even if at first it looked somewhat incredible. The matter being mentioned in our Medical Society after my return, I started the question, whether any objections could be offered to my making essays with this new method of cure, when these were made only on individuals suffering from constitutional syphilis. My colleagues agreed with me as to the propriety of

---

* From Auzias Turenne's *Cours de Syphilization*, p. 14, we see that he too had experimented on men, but this he had not previously published, and it was therefore unknown to Sperino.

the experiments under the condition named, and, accordingly, I began my practical investigation of the subject of syphilization as soon as I was able to obtain some inoculable viris, which was not, however, till the month of October, 1852, from which time my first inoculations are dated. From that up to the present date, I have syphilized more than 200 individuals. Concerning my method of syphilization, and the results obtained from it, I shall now proceed to give a short account.

As I have already said, I only consider syphilization indicated where there is *constitutional syphilis;* because I am not quite certain that the primary syphilis (thus introduced) will not occasion a constitutional syphilis; and I have no right to bring into the body a virus that was not there before.

I begin the syphilization by taking the matter from a primary ulcer, but not caring whether it is simple or indurated. One may very well syphilize with matter from either, and nothing perhaps will better prove that the supposition as to the complete difference, which latterly (by Ricord, Clock, Basseron, etc.) has been seen to exist between the matter from the simple chancre and from the indurated chancre, is groundless. However, it is not my intention to say that all syphilitic virus is of the same nature. On the contrary, experience has taught me that there is a great difference in the intensity of the matter, and also in its syphilizing virtue. The virus taken from a primary ulcer I inoculate in the same manner as I inoculate the vaccine; and if I wish as much as possible to avoid great ulcers and cicatrices, I make the first inoculations in the sides of the trunk. Hitherto I have generally put three in each side. After three days there will be pustules; from these I now take matter for making a new inoculation, which I put at a distance of two-third inches from the first one. In this way I continue inoculating every third day, always taking the matter from the last pustules, as long as it is possible with this matter to produce *any* effect.

In observing the series of inoculations that are made, we will find that the ulcers, after the first inoculations, are the largest ones; then those of No 2; and thus they diminish after the order in which they are made, until at last there only come aborting pustules. One might believe this to be occasioned by the intensity of the virus having diminished; but we will be convinced that the reason is in the inoculated individual himself, by taking the virus that is of no more effect upon him and applying it to another individual, when it will be seen to produce great pustules.

The first virus not making any more effect when the individual has grown inured to it, matter from another individual will, however, prove inoculable. But the first pustules and ulcers that come forth after this second matter will not nearly be so large as those from the first matter. If we will now take a third matter, it will also bring forth pustules and ulcers, but still smaller ones, and still fewer in number, than those from the second matter; and at length we come to the point, when no matter applied in the sides produces any effect.

Still, however, there is yet no general immunity. If I were to inoculate the thighs with a new matter, or with the last matter that no longer produces any effect in the sides, I am always sure to see there a positive effect. I am generally accustomed to inoculate at the same time on the thighs and the arms; and

I go on in the same manner as I have described on the sides, until also on these places I come to the point when no matter that I can obtain will have any effect. If it is of no importance whether or not I call forth large ulcers and cicatrices, I begin the inoculations on the arms and the thighs. On the arms, the pustules and ulcers will be of the same size as those on the sides; but on the thighs they will be larger, and the series of inoculations may be a very long one. Having, by continued inoculations in these places, attained the result of no longer producing any effect, if we were now to try to inoculate on the sides, we would have no effect at all, or at least a very insignificant one.

From these results of syphilitic inoculation, I think there are four points that are interesting to observe, and that are of great importance as physiological facts:—

1. The artificial chancres on the sides and on the arms are always smaller than those on the thighs, and the series of inoculations shorter.

2. By continued inoculation the ulcers always become less and less, until at last the inoculation gives a perfectly negative result.

3. The inoculated individual grows inured to one matter, but is still receptive to another, yet in a lower degree; and again, to a third one, but in a still lower degree; and so on till no further effect is produced by any matter.

4. Immunity having been obtained on the sides and on the arms, it will still be possible to have a rather long series of inoculations on the thighs; however, we will always be able to observe plainly the influence of the inoculations previously made on the sides and on the arms, inasmuch as the ulcers that come forth on the thighs will not be so large, and not so many, as when the inoculations have been commenced on them. If one has begun by inoculating the thighs until immunity has been produced there, the effect of the inoculations subsequently made on the arms and on the sides will be comparatively insignificant.

All these phenomena are constant; they will not be seen in one individual, and missed in another; they will always show themselves. We have here an invariable law of nature.

We see that the inoculations make a general effect upon the organism. This is most decidedly shown to us by the three results last mentioned. If the organism was not influenced from the absorption of the inoculated syphilitic virus, we would be unable to explain to ourselves the fact, that the later inoculations are always less efficacious than the earlier inoculations. Soon after the introduction of syphilization, the opinion was propounded, that all depended upon the derivative effect of the artificial ulcers, which were only to be considered as little fountains. Such an opinion was rather likely to be uttered at that time, but by repeating it at the present, it only proves a perfect want of acquaintance with the subject of syphilization. It is impossible to keep such an opinion after having followed the progress of one single syphilization.

But, can it perhaps be the case, that the organism, by the repeated use of general derivative remedies—such, for instance, as tartar emetic—grow inured also to this remedy? From what I have heard, however, from those who have tried to heal syphilis by such a derivation, this has not been the case.

Now, if the diminution by degrees of the ulcers of inoculation in one or other part of the body, should not be found sufficiently evident, I should think the fact that, after the immunity has been produced by inoculations on one part of the body, the receptivity is less on another part, does not permit any doubt as to an absorption having taken place. It is evident, that the state of the organism is no longer *the same* as before the inoculation. But what may not be doubted is explained wrongly by those who will not observe for themselves, or will observe with prejudiced opinions. For them it is of no use even to prove that, as I have said before, by continued inoculations, we come to the point when no matter will any longer produce any effect. They affirm hardily that there does not exist any immunity, and that all that has been done is mere fancy. They see very well, that, if they were able to prove this, it would be proved also that there was no probability as to there having been any absorption of the virus, or as to an altered state having taken place in the organism. If by continued inoculation the result is immunity to the syphilitic virus, syphilization obtains some analogy with vaccination, and with the acute exanthemata in general. This *a priori* looked absurd; and some did not hesitate to deny its possibility, before they were convinced by personal observation. Only after having uttered this prejudice they began to experiment; and then, of course, the experiments proved what had been found out in the study. It was forgotten that these experiments might be repeated by others, and that such incorrect observation would, in a short time, be discovered. The formation of more than 2000 chancres, without the production of immunity, has been spoken about; but it has been communicated to me that, in this case, the pustules, as they came forth after the inoculations, were always cauterized, so that, if this is true, they thus prevented the absorption, and changed the syphilitic ulcers into common ulcers; and, I have no doubt, they might continue inoculating in this way to all eternity, without effecting any immunity. Further, they have, where the inoculation, undertaken in the usual way, the same as in vaccination, did no longer give any result, sent the syphilitic virus far in under the skin, and filled the opening with cotton, or they injected the virus into the cellular tissue. There is nobody, I suppose, who doubts of this having caused a suppuration; but nobody will doubt, also, of this being the way to call forth a suppuration, by applying any purulent matter whatever in a similar manner.

With such facts they have endeavored to annul observations made with the greatest exactitude, and repeated in several hundreds of cases.

It would not, however, be possible to make the question of immunity doubtful, even for ever so short a time, if there were not circumstances that came to the assistance of inexact and superficial observers; and such are certainly to be found.

I have before observed, that when an individual has grown inured to the matter first applied, still a second, a third, a fourth, and perhaps a fifth matter will give a positive result; the effect, however, of every subsequent matter being fainter than that of the preceding, and the ulcers becoming less and less, until at last there are no more characteristic ulcers,—only insignificant excoriations. And while, in the first inoculation, the matter could be brought through scores of repetitions, we will not at last with a new matter be able to produce more than one single ulcer by repeating the inoculation with it. Now, it happens in some syphilized individuals, that we may several times inoculate with a new matter and only bring forth small pustules, which will soon dry, and which may truly be named abortive. If we take the small quantity of matter they sometimes contain, and inoculate the same individual with it, we shall have a perfectly negative result.

To those who observe with exactitude, these facts are very easily explained; it is only those who have not studied syphilization, and followed the steps of the process, who think it wrong to speak of immunity as long as a new inoculation will perhaps give a positive result.

This, after all, is only a dispute about words. I have employed the word immunity, because it was employed by Auzias Turenne, and Sperino, and I am no friend of making new words when I can help myself with old ones; but if, after what I have explained, this word should be found to be incorrect, it is no matter to me if they chose another.

The question is, whether the organism, after the completion of the syphilization, is still in the same state in respect to the syphilitic virus as before; and to this, everybody who has observed with judgment, will answer—"No;" and the difference between the condition of the organism in respect to its immunity to the syphilitic virus, before and after syphilization, is so great, that I assure those gentlemen who have opposed this fact, and denied its existence, that within a short time, when more persons have deigned to examine the important question more closely, and make experiments, they will be left alone with their opinions. Whether this alteration of constitution proves that the syphilitic virus no longer exists in the organism at all, I dare not say with certainty; on the contrary, we see from facts I am going to mention afterwards, that the virus exists in the body, at least for some time after immunity has been obtained,—but how long, is still a question.

There is yet another question that has often been put, viz., How long does the immunity last? Does it last for the whole life, or only for a time,—and then for how long? We know that the acute exanthemata generally do not attack the organism more than once; that a successful vaccination preserves for many years; and that variolæ, if they come after vaccination, are considerably modified. Whether there exist similar conditions after syphilization, we must confess we are yet ignorant.

I have been reproached for not having made experiments in that direction. But when I had cured my patients, I considered that I had no right to inoculate them anew; just as much as I do not think it right for me to inoculate healthy persons with the syphilitic virus. In some cases, however, especially in those persons who were suffering from incurable maladies, cancer, etc., and whom I had syphilized experimentally, I have made new inoculations some time after having finished the syphilization. The result of these has been absolutely negative; or, perhaps, after some time there have come forth very small pustules and ulcers, the matter of which could only be propagated through one single, or a very few ulcers more. If I made the new inoculations after the lapse of months, I could only transmit the effect through some more sets of ulcers; but there was never the least comparison between this and the earlier effect of the inoculations; the ulcers never became extended; and the *altered* state to which the organism had been brought by the earlier inoculations was so clearly proved that it was impossible to misjudge it.

There are two questions that have often been confounded, viz., the first concerning the existence of immunity; the other concerning its duration. I positively insist upon these two questions being entirely separated from each other. It is an indisputable fact, that by repeated inoculations, we reach the point where we cannot produce any more effect by the inoculation. This fact is equally remarkable, and equally important, if the immunity attained were only to last for a day, or even for an hour. This physiological fact merits our whole attention, without any regard to the duration of the state attained. If the immunity is of no duration, then it will only prove that it would be possible again to be attacked by syphilis. But as he only gets syphilis who exposes himself to it, the cessation of immunity to this malady will not be of the same importance as it would be in the case of the acute exanthemata, which we get in spite of ourselves. I do not, however, by any means intend to say that the insignificant series of inoculations that may possibly be made after the completion of the syphilization proves that the immunity has ceased, and that a new general infection is possible. But as the new series of pustules and ulcers is in every respect very insignificant, so I think the syphilis that might be caught in the ordinary manner, would also be of no great importance, and, in my opinion, would not occasion any evolution of constitutional syphilis. But it is far from my meaning to anticipate experience; it alone will be able to decide with certainty this question; and even if the effect of syphilization should not always continue for the future, we must confess, I think, that its effect upon the present forms of constitutional syphilis is so great and beneficial, that we may be satisfied with that alone.

I now proceed to speak of the effect of syphilization upon constitutional syphilis. And, in the first place, I shall make the observation, that we must make a great difference between those cases which have not been treated previously and those which have been treated, especially if they have been mercurialized. For only in the cases where no mercury has been used, does syphilization show its real and uncomplicated effect.

When we make the inoculations in the manner above described, we sometimes, in from four to six weeks, observe some influence for the better on the symptoms present; but this improvement is generally of no duration;—fresh eruptions will appear, and the whole phenomena will seldom have disappeared till after three or four months. As to the time required for the treatment, the nature of the phenomena present is of importance. From the experience I have gathered in about one hundred cases, it seems evident that, where the skin is occupied to no great extent by the syphilitic eruption, the effect of the inoculations, in respect to the local production of pustules and ulcers, as well as to their influence upon the organism in general, is more regular. In the more unusual cases, where the skin is entirely occupied by the eruption, especially in the papular form, the inoculation is often difficult. The first series of inoculations generally go on in the usual way—it is not generally, however, very long; but new matter, although employed for some time, makes little or no impression. Under these circumstances, we must wait, if I may so express myself, till the first series has produced its effect upon the organism, and the eruption consequently has begun to disappear; then we observe fresh inoculations have better effect, and the disease diminishes accordingly. In consequence of the slow effect of the inoculations in the latter class of cases, the treatment will perhaps last even six months, or more. This is a long time; but if an individual, suffering from severe constitutional syphilis, can be assured of *certain* cure in about six months, I think it must be allowed that this time is well employed. Immunity having been produced, and the symptoms existing at the beginning of the treatment having disappeared, new symptoms generally show themselves, especially sores on the mucous membrane of the mouth, or a fresh exanthematic eruption, or iritis. These new phenomena disappear without any treatment being directed against them; even the most intense iritis will pass away completely without our meddling with it at all. I let the patient remain in a light room, without any screen for the eye, or go out, if he chooses. I allow him to eat and drink what he likes, and employ neither internal nor external medicaments; and the result, in all the cases I have so treated, has been quite satisfactory. The inflammation ceases, the eyeball grows movable, and the patients have never had their vision impaired. In some cases, small effusions of lymph have taken place, but they have occasioned no marked disturbance of sight.

These new phenomena may also occur during the process of syphilization; I then treat them just as when they come afterwards;—I take no notice of them whatever.

Syphilization may be practiced at any age. I have treated very young children after the same method, and with the same happy results as in grown-up persons. In children, the pustules and ulcers are much smaller, and their receptibility to a great number of chancres very much less; the time required for treatment, also, is often shorter.

From the results I have hitherto obtained from syphilization in those who have not been previously mercurialized, I venture to affirm, that *in no disease have we a more certain method of cure.* When to this is added, that the patients, during the treatment, as well as afterwards, are quite comfortable, that they may eat and drink whatever they like, and attend to their business, and even, if there is any suffering from the constitutional vice, the state of health is improved, I think it should satisfy all the expectations we by any means can form of it.

The circumstances are not the same when mercury has been used before. Having tried syphilization in about one hundred cases of this sort, the following are the chief results of the treatment:—

1. The local effect of the inoculations is not so uniform as when mercury has not been used; it may even cease entirely after some time, and not begin again till a few doses of iodine have been given.

2. The effect upon the syphilitic symptoms present, also, is not so uniform.

3. Syphilization does not seem to have any, or at least any considerable, influence upon affections of the osseous tissues and derangements of the nervous system, occurring after syphilis.

4. One is often obliged to combine the use of iodine with syphilization, in order to obtain a complete cure.

5. Relapses are not unfrequent after syphilization in individuals previously mercurialized.

6. These relapses, however, will never have a form worse than that which existed before the syphilization: they will always, so to speak, be a fraction of the earlier affection.

7. The state of health always improves during the syphilization.

It will be seen from the above-mentioned details, that, although it is often impossible to do everything with syphilization in those who have been previously treated with mercury, yet even in such cases we obtain very much better results than by any other treatment whatever. It is a duty, therefore, in these cases also to use this treatment. But it is not, as some persons have believed, especially in these circumstances that syphilization ought to be employed—only when all other remedies have failed. Syphilization is the remedy against syphilis, but not against depraved syphilis—that produced by improper and pernicious remedies, especially mercury. It is the first and not the last remedy in constitutional syphilis; this point we must insist on, in order that it may be understood that it is not necessary to empoison our patients with mercury before proceeding to cure them.

Perhaps I ought to demonstrate more fully my assertion as to the insufficient and pernicious effect of mercury on syphilis; but in an Edinburgh journal it will be unnecessary, as I know that my opinion of this remedy is approved in Scotland.—*Edinburgh Medical Journal*, April, 1858.

3. Excision of the Tongue for Cancer. By *James Syme, Esq., Professor of Clinical Surgery at the University of Edinburgh.*—There are few diseases more painful to suffer or more distressing to witness than cancer of the tongue. The concurrent testimony of all experienced practitioners is decidedly unfavorable to operative interference by removal of the diseased part from its never affording permanent relief, and generally exciting, instead of retarding, the morbid progress, while all the means of palliation are extremely uncertain and inefficient. In circumstances so desperate any practicable measure affording a reasonable prospect of relief would certainly be warrantable; and under this impression I have lately tried the effect of extirpating the whole organ. As the disease frequently exists for a long while without extending beyond the tongue, and yet rapidly reappears after the affected part has been cut freely away, there seemed reason to think that if the whole texture showing this disposition to morbid action were removed the patient might escape future trouble. Some reports had reached me of this having been done by cutting into the mouth under the chin, and then completing the operation by means of either a knife or the écraseur. But feeling assured that it was impossible to accomplish the object fully and satisfactorily through the imperfect access thus afforded to the root of the tongue, I resolved to divide the jaw at the symphysis, and then draw the two halves aside so as to get room for accurate dissection and ligature of the vessels.

G. S——, aged forty-seven, a shoemaker, was admitted into the hospital on the 11th of November, 1857, suffering under extensive cancer of the tongue, which was diseased throughout its whole extent, except toward the root. He stated that only five months had elapsed since the commencement of his complaint, or, at all events, since his attention was first called to it. There was no glandular swelling, or any other sign of disease.

On the 9th of December I made an incision through the lip, and extended it down toward the os hyoides, then sawed through the thick part of the symphysis, and completed its division by cutting pliers; next had the two halves held aside while I dissected backward, so as to cut and tie the lingual arteries near the cornu of the os hyoides, and finally detached the tongue closely from the body of this bone. The edges of the wound were then brought together, and the patient walked stoutly to bed. Food was supplied by the injection of milk and beef-tea through a tube introduced into the œsophagus, and everything went on so favorably for several days that I entertained no doubt as to the patient's recovery. But on the fourth day he began to complain of uneasiness in his chest, had a quick pulse, and became feeble. These unpleasant symptoms rapidly assumed a still more serious aspect, and terminated fatally on the seventh day. On dissection, the wound, larynx, and trachea were found in a satisfactory state; but the lungs were thickly interspersed with small indurations indicative of recent inflammatory action.

In considering this case, I felt at some loss to determine whether the fatal issue had necessarily resulted from the operation, or was owing to circumstances that admitted of prevention. The patient was ascertained to have been addicted to habits of intemperance. The wound had been healed throughout its whole extent, so as to leave no room for the escape of discharges from the fauces, and from an early period after the operation experiments had been tried on the power of deglutition with the effect of exciting a great deal of sputtering and cough. It seemed not improbable that, but for these unfavorable circumstances, the operation might have proved successful, and, therefore, although certainly having no great desire to repeat it, I did not feel entitled to decline doing so should a proper case present itself.

A few weeks ago I received an application from the hospital at Northampton to admit a patient, who appeared a very suitable subject for the purpose. He was fifty-eight years of age, a remarkably respectable, healthy-looking man, without the slightest sign of glandular disease. He had suffered for nearly six years from disease of the tongue, and in the early part of last spring had undergone an operation by ligature in the Northampton Hospital. In consequence of the disease returning, he afterwards went to London, (Guy's Hospital,) where no operation was performed, but some strong applications were employed. He then went home, with the tongue greatly swelled, and diseased throughout its whole extent, except toward the root, where the texture retained its ordinary characters, so far as could be ascertained by the finger.

On the 31st of July I performed the operation precisely as on the former occasion; but instead of closing the wound throughout its whole length, left about an inch of it open at the lower part, and inserted a piece of lint to prevent adhesion. I also directed that no food should be given except by injection through a tube. Again things went on favorably for a few days, and again I entertained sanguine expectations of success; but on the evening of the third day, symptoms of the same alarming character as in the first case showed themselves, so that next day I saw there was no hope, and felt prepared for the fatal result, which took place on the following day. On dissection, the air-passages were again found free from signs of disease until they reached the lungs, where there were extensive indications of recent inflammation.

In both of these cases it was manifest from inspection of the mass removed that no other operation could have accomplished complete extirpation of the tongue, and unless this were done, no better result could be anticipated than from the partial removal, which has always been found so useless and objectionable. On the other hand, I felt satisfied from the similarity of the symptoms and result, that the operation, if not certainly fatal, must be one of extreme danger; and as even in the event of success, the permanency of relief would still admit of question, I think there should be no hesitation in deciding against the repetition of this procedure. In promoting the progress of surgery, it is hardly of more consequence to determine what is expedient than to ascertain what is not expedient; and I venture to hope, that the experience now related may not prove useless by saving others from the disappointment which I have myself experienced.—*Lancet* August 14, 1858.

[In addition to the cases of Mr. Syme, we extract the following letter from the Paris Correspondent of the *Medical Times and Gazette* for September 4th, 1858:]—

The following short notes of a case in which M. Ricord removed, at the Hôpital du Midi for lingual cancer, the entire organ as far as that was possible with the écraseur alone, may be of some interest to our friends at home at a time when the attention of the medical public is directed to the propriety or impropriety of the entire removal of this organ for cancer by so great an authority as Professor Syme, who, having lost his two cases in precisely the same manner as M. Sedillot lost the first patient upon whom he had performed exactly the same operation some years ago, and who, failing with all his skill and boldness to obtain even a temporary success, comes forward like a courageous gentleman, and holds up his cases of failure as a beacon to the profession at large; and now openly avows his concurrence with the testimony of all experienced practitioners as to operative interference being decidedly unfavorable in these cases, the removal of the diseased part never affording permanent relief, and generally exciting instead of retarding the morbid progress.

About the month of March of the present year, or at any rate, at the time of the last fall of snow, M. Ricord had in his wards a patient with cancer of the tongue, who had been variously treated by caustics, but was at that time in such a condition that his very existence was a misery. He was totally deprived of speech, and had completely lost the faculty of taste, excepting, as M. Ricord remarks, "le goût de son propre fruit." In such a state of suffering was this poor fellow, that he allowed the rather unwilling operator no rest until he had performed his duty.

This was effected in the following manner: the tongue seized with the vulsellum, and drawn forcibly forward, a strong curved trochar was passed from side to side, (or, speaking of the tongue, from edge to edge,) and as far back as possible; the chain of the écraseur was then passed behind the trochar, and the

"écrasement" effected very slowly, one notch per minute; however, in spite of this extreme slowness of the crushing process, the operation was followed by some amount of hemorrhage; this, nevertheless, was arrested by cramming the mouth with snow-balls soaked in the perchloride of iron.

All went on well during the first fortnight, so that at the end of that time he had not only recovered the faculty of taste, so as to be able correctly to distinguish the difference between cabbages and lentils, and the like different dishes forming the fare more or less sapid and sumptuous of the hospital; but he had likewise become the orator of the ward, speaking French with sufficient ease and fluency, although he would probably have been somewhat puzzled to pronounce the pure linguals of the English language.

About this time he was exhibited to the assembled Academy; and presenting in the submaxillary region some slight induration with glandular engorgement, M. Velpeau pronounced his opinion that this was evidence of a return of the affection. This opinion, however, was combated by M. Ricord upon the grounds that the glandular engorgement was more easily and hopefully explained by the supposition that it depended upon a retention of the salivary secretion, owing to the ducts having been included in the operation; he inclined to this opinion, inasmuch as the engorgement made its appearance very suddenly and equally on either side. The diagnosis of M. Ricord proved the correct one; the engorgement, after having attained an enormous extent, gradually subsided. But now arose another and unexpected accident; as cicatrization progressed the contracting cicatrix laid a tax upon all the surrounding parts; what little was left of the tongue, previously mobile, and capable of modulating the vocal element, was so drawn upon and forward that it became by degrees immobile, again depriving the patient of vocalization. But this was not all; the still contracting cicatrix now began to affect more distant parts; deglutition, from less and less facile, became more and more difficult, until we fear that inanition might have subsequently become a serious, and even fatal complication, had not the case been suddenly cut short by a latent attack of pneumonia at the commencement of the past month. The pneumonia was not suspected, owing to the absence of expectoration and other outward and visible signs. This may be excused, owing to the fact that all attention was directed to the obviation of the imminent ultimate result of the interference with the function of deglutition.

The necroscopy discovered no evidence of cancerous return; the salivary glands, pharynx, and œsophagus were perfectly normal; the mucous membrane of the larynx slightly indurated, this organ otherwise healthy; the lungs a perfect specimen of red hepatization; other organs normal.

From the above case we should not decide formally with Mr. Syme in his veto against the future performance of this operation, but we would reiterate the opinion and views of your Scottish Tourist. For the future we should dispense with the indispensable ligature left in order to prevent the patient from swallowing and strangling himself with the remainder of his tongue, and we should, in searching for the cause of this constant pneumonia, seek to obviate the accidents so imminent in this case.

On the other hand, we have other cases of a more happy termination. The other day, (August 9th,) for example, we saw a case in which more than twothirds of the tongue had been removed by the écraseur two years previously; the patient presented a specimen of even ruddy health, although the tumor removed by M. Chassaignac was reported as cancerous.

We have seen M. Maisonneuve remove the entire tongue, after having previously divided the lower jaw at its symphysis, by means of a chain-saw, and completing the operation either by means of scissors or the écraseur. He looks upon hemorrhage as quite a secondary consideration. The presentation of patients having undergone the entire removal of the tongue before l'Académie de Médecine is a sufficient guaranty that the operation is not in itself necessarily fatal. And also from our own observation we can assert that in the cases of M. Maisonneuve, the operations seen by us were, we may say, well supported, although we have now lost sight of the patients, and have no note of the ultimate results. We have noticed considerable difficulty in keeping the divided surfaces of the jaw in direct apposition; and in order to obviate this inconvenience M. Sedillot suggests that the maxilla be divided by two oblique lines meeting in the centre, so that one end fitting into the other they may be of mutual support.

4. DISPLACEMENT OF THE TESTICLE INTO THE PERINÆUM; PLASTIC OPERATION UNSUCCESSFUL; CASTRATION. Under the care of *R. Partridge, Esq.*—Benjamin S., aged twenty-four, was admitted into King's College Hospital on April 30th, under the care of Mr. Partridge, on account of displacement of the left testicle into the perinæum. It appeared that he was a soldier in the Horse Artillery two years ago, and that he was thrown forward, while riding, on to the pommel of the saddle, and received the injury for which he was admitted. He fell from his horse, and vomited for some minutes. Next morning he observed a swelling in the perinæum about the size of a hen's egg, and so painful that he could hardly move about. He then went into the military hospital, and was under treatment for about three months, but without benefit; the testicle only gliding further backward in the perinæum till it reached its present position—about an inch in front of the anus. The testicle had diminished in size, and was smaller than the right. The vas deferens could be distinctly felt. He sometimes had difficulty in passing urine; but at others it flowed easily. He was quite unable to work.

May 19th.—An operation was attempted for the purpose of replacing the testicle in its position. The patient was placed in the position for lithotomy; a transverse fold of skin was pinched up over the testicle in the perinæum, and divided. A band of fibrous tissue which restrained it (perhaps the remains of the gubernaculum) having been divided, the testicle was pushed up toward, and as much as possible into, the scrotum, and retained in its place by deeply placed subcutaneous sutures, and by a pad and bandage in the perinæum.

June 1st.—The wound was gradually healing. The patient complained of pain in the course of the spermatic cord.

June 14th.—It is noted that the testicle had gradually slipped back into its old position, and that the patient feels as much pain there as ever. As it seemed hopeless to attempt again the reduction of the testicle, it was determined to remove it. Accordingly, on June 19th, the patient was again brought under the influence of chloroform, and an incision was made on the spermatic cord. This was then taken up, a ligature placed on it, and the testicle removed. The ligature came away in a few days, and the patient is now convalescent. The testicle was, we believe, examined after removal, and found atrophied to some extent from pressure and injury, but otherwise healthy.

*Remarks.*—We have not met with any other instance of this injury—dislocation, as it may be termed, of the testicle. It is, in fact, one of which it is difficult to conceive the possibility in a perfectly natural condition of the testicle and its appendages, on the one hand, and of the scrotum, and other soft parts, on the other; and it was on this account that, in this case, some irregularity was suspected in the original conformation of the parts, Mr. Partridge having expressed his opinion that the gubernaculum had had an unusual attachment which predisposed the testicle to pass down below its natural level. One thing seems plain, viz., that the testicle must have been in its usual place just before the accident, otherwise the patient could hardly have done duty as a horse-soldier. The treatment followed was undoubtedly judicious, and would probably, if another such case should occur, be more successful. In this the communication between the scrotum and perinæum appeared to be too free to allow any chance of closing it by operation, and the testicle was producing an amount of misery in its new position which rendered it useless to think of leaving it there. —*British Medical Journal,* July 10, 1858.

